# The potential for automated question answering in the context of genomic medicine: an assessment of existing resources and properties of answers

**DOI:** 10.1186/1471-2105-10-S9-S8

**Published:** 2009-09-17

**Authors:** Casey Lynnette Overby, Peter Tarczy-Hornoch, Dina Demner-Fushman

**Affiliations:** 1 Department of Medical Education & Biomedical Informatics, University of Washington, Seattle, WA, USA; 2 Department of Pediatrics, University of Washington, Seattle, WA, USA; 3 Department of Computer Science & Engineering, University of Washington, Seattle, WA, USA; 4 Lister Hill National Center for Biomedical Communications, National Library of Medicine, NIH, BHHS, Bethesda, MD, USA

## Abstract

Knowledge gained in studies of genetic disorders is reported in a growing body of biomedical literature containing reports of genetic variation in individuals that map to medical conditions and/or response to therapy. These scientific discoveries need to be translated into practical applications to optimize patient care. Translating research into practice can be facilitated by supplying clinicians with research evidence. We assessed the role of existing tools in extracting answers to translational research questions in the area of genomic medicine. We: evaluate the coverage of translational research terms in the Unified Medical Language Systems (UMLS) Metathesaurus; determine where answers are most often found in full-text articles; and determine common answer patterns. Findings suggest that we will be able to leverage the UMLS in development of natural language processing algorithms for automated extraction of answers to translational research questions from biomedical text in the area of genomic medicine.

## Introduction

Translational medicine attempts to connect basic research to patient care, and is often referred to as "bench to bedside." For example, knowledge gained from studying genetic disorders may aid in providing more personalized care for patients [1]. This type of knowledge is reported in a growing body of biomedical literature containing reports of genetic variation in individuals that map to medical conditions and/or response to therapy. Current practice of medicine is often reactive, where treatment does not occur unless one becomes sick. Research institutes are envisioning a shift in current medical practices to a more predictive, personalized, preventative and participatory (P4 Medicine) model [2,3]. Particularly in the context of genomic medicine, we may be able to utilize knowledge reported in biological literature to facilitate the development of a model for practicing P4 medicine.

In the area of genomic medicine, we may ask the question "given the vast amount of biomedical literature, how do we determine an answer to a translational medicine-related question?" As a preliminary step towards answering this question, we aim to determine whether existing question answering methods and biomedical informatics resources will be useful for identifying and extracting answers to translational research questions from biomedical text. The resources we wish to investigate include the 2008AA UMLS Metathesaurus and the MetaMap program.

The UMLS initiative [4] of the National Library of Medicine aims to unify the medical vocabularies of different medical knowledge sources, the UMLS Metathesaurus is a lexical framework for this integration. The MetaMap Program (MM) [5] is a program that finds UMLS concepts in the text and may be useful for mapping biomedical text to concepts in the UMLS Metathesaurus.

In the context of genomic medicine, the objectives for our study were to: 1) Evaluate the UMLS Metathesaurus coverage of translational research terminology; 2) Assess the ability of the MM program to map translational research terms from biomedical literature to the UMLS; 3) Determine where answers to translational research questions are most often found (title, abstract, conclusion, etc.); and 4) Determine common answer patterns to translational research questions.

## Related work

In our research, we want to determine whether NLM tools will be useful for extracting answers to translational research questions from full-text articles in genomic medicine. Information Extraction (IE) aims to provide a user with facts and knowledge in an easy to understand fashion. Our evaluation of answer patterns will be useful for future development of IE algorithms. Related to this endeavor is the Repository for Informed Decision Making (RIDeM) project [6] that provides access to information needed to support clinical decision-making. The current prototype provides key facts relevant to a clinical question or a patient's record by matching clinical concepts extracted from clinical text and salient points extracted from MEDLINE abstracts. Clinical questions are captured using a PICO-based framework [7], and MM and the UMLS are utilized to extract facts from MEDLINE abstracts. Unlike clinical question answering, providing answers to questions in the practice of genomic medicine is a relatively new research area.

The interest to genomic information retrieval and question answering was reflected in the Text Retrieval Conference (TREC) Genomics Track evaluations [8]. The 2006 and 2007 evaluations focused on answering genomics research questions by extracting answers from full-text biomedical literature [9,10]. Questions from the 2007 TREC Genomics Track evaluation were derived based on biologists' information needs, and answers were lists of named entities of a given type. We use the extracted answers from the 2007 TREC Genomics Track evaluation as part of our gold standard dataset in the presented research.

## Methods

We had four objectives in the genomic medicine care context: *Objective 1 *– Evaluate the UMLS Metathesaurus coverage of translational research terminology; *Objective 2 *– Assess the ability of the MM program to map translational research terms from biomedical literature to the UMLS; *Objective 3 *– Determine where translational research questions are most often found (title, abstract, conclusion, etc.); and *Objective 4 *– Determine common answer patterns to translational research questions.

### Common method across objectives

We used the 2007 TREC Genomics Track evaluation data set [11] as our gold standard. Of the 29 questions explored in this evaluation, we were able to identify four questions that were translational research related. These questions include the following:

• <201> What [MUTATIONS] in the Raf gene are associated with cancer?

• <216> What [GENES] regulate puberty in humans?

• <218> What [GENES] are implicated in regulating alcohol preference?

• <224> What [GENES] are involved in the melanogenesis of human lung cancers?

For each question we had a set of answer passages, full-text (FT) documents for each of these, and associated answer concept(s). Biologists participating in the 2007 TREC Genomics Track evaluation assigned answer passages to the appropriate answer concepts. An example answer passage and associated answer concept is as follows:

Answer Passage: "Genetic heterogeneity of constitutively activating mutations of the human luteinizing hormone receptor in familial male-limited precocious puberty"

Answer concept: "LUITENIZING HORMONE RECEPTOR (LHR) GENE"

The gold standard data set was used differently for each objective. For the first objective, we searched the UMLS for answer concepts from our gold standard data set. UMLS concepts that matched, or were synonyms of, our gold standard answer concepts were used for assessing the accuracy of MM in our second objective. Our third objective required that, for each gold standard FT document, we search for, and note, the location of associated answer passages. For our fourth objective, we reviewed answer passages for each of our four questions and identified common answer patterns. More details on the gold standard data set follow.

### Methods for objective 1

For our first objective, we evaluated how well the UMLS covers translational research terminology. For each gold standard answer, we manually identified two types of matches, exact concept matches and partial concept matches. A concept in the UMLS is considered an ***exact match ***if it exactly matches, or is a synonym of, the gold standard answer. For example, if our gold standard concept is ADH and we identify Alcohol dehydrogenase as a concept in the UMLS, we would consider this to be an exact match because the two concepts are synonymous. A concept is considered a ***partial match ***if an exact match does not exist in the UMLS, but the major concepts in a multiword concept exist. For example, if our gold standard concept is GABAA RECEPTOR, #1 SUBUNIT, although there is no exact match in the UMLS, we would identify the UMLS concept GABA-A Receptor as a partial match. In our next objective, we compare our UMLS match results to our MM retrieval results.

### Methods for objective 2

For our second objective, we evaluate how well MM maps translational research concepts from answer passages to the UMLS Metathesaurus. For all questions, the following UMLS semantic types were extracted as potential answers: GENE OR GENOME; GENETIC FUNCTION; AMINO ACID, PEPTIDE, OR PROTEIN; RECEPTOR; NUCLEOTIDE SEQUENCE; AMINO ACID SEQUENCE; MOLECULAR SEQUENCE. Lexicon-based methods (such as MM) require the inclusion of all of these UMLS semantic types for two reasons: 1) use of ambiguous names by paper authors, and 2) UMLS multi-word sense coverage. An example of the prior is use of "BRAF" for both the BRAF gene [GENE OR GENOME] and BRAF protein, human [AMINO ACID, PEPTIDE, OR PROTEIN]. Both terms are in the UMLS, but fairly sophisticated context understanding is needed for disambiguation. As an example to illustrate UMLS multi-word sense coverage, even if an answer explicitly mentions "cannabinoid receptor gene," this concept is not covered in the UMLS, and MM will break it up into cannabinoid receptor [AMINO ACID, PEPTIDE, OR PROTEIN; RECEPTOR] and Gene (Genes) [GENE OR GENOME].

In order to measure MM's performance, we used a method similar to that of a previous study where two types of matches were identified for retrieved concepts [12]. We identify each match as an exact or a partial match based on matches found in the first objective. A concept is considered an ***exact match*** if MM identifies a concept found to be an exact match in the UMLS. We consider a concept to be a ***partial match*** if the MM identifies a subset of a multiword concept found to be a match in the UMLS. For example, if our exact match in the UMLS is GPR54 gene and MM identifies G Protein-Coupled Receptor Genes, we would consider it to be a partial match. In addition to identifying exact and partial matches for our MM results, we also did so for our baseline entity extraction method. Our baseline entity extraction algorithm retrieves all gene specific word shapes [13]. When the baseline algorithm extracted a word shape that was synonymous to our gold standard answer concept, it was considered an ***exact match***. For example, if our answer is LUITENIZING HORMONE RECEPTOR (LHR) GENE, and our baseline algorithm extracts "LHR", we would consider this an exact match. We considered our baseline data term to be a ***partial match ***if it was not an exact match, but matched a content-bearing word in a multiword gold standard concept. For example, if our answer is B-RAFV599E, and our baseline algorithm extracts "B-RAFV," we would consider this to be a partial match.

We compared our MM retrieval results to the results of the baseline set to show how the controlled vocabulary-based MM algorithm compares to a baseline algorithm. This comparison is accomplished by calculating *precision *and *recall *measures. ***Precision (MetaMap/baseline) ***was calculated as the number of gold standard individual concepts with which our method found matches, divided by the total number of concepts that the method identified. ***Recall (MetaMap/baseline) ***was calculated as the number of gold standard individual concepts identified by the method, divided by the total number of gold standard individual concepts. Details about our comparison between the MM and baseline algorithms may be found in the **Result for objective 2 **section. In our next objective, we were interested in determining where answers to translational research questions are most often found in biomedical text.

### Methods for objective 3

For the third objective we looked at where our gold standard answer passages appeared in the associated FT documents. Specifically, for each answer passage, we made note of in which of the following FT section answer concepts appeared: title; abstract; introduction; discussion; results; references; no section titles; or other. An answer may be included under "no section titles" if it is located in the body of an article that doesn't have section titles. Section "other" includes all section titles that were not listed above. We then tallied up and reported on the locations of our answers by FT section. In our final objective, we looked at each gold standard answer passage and recorded answer patterns for each.

### Methods for objective 4

For our fourth objective, we determined common patterns that exist in answer passages. To do so, we first constructed a table of patterns for each of our four questions. An example answer pattern is "MutationType of GeneName OBSERVED in [SubjectType] with PrimaryCondition." More details about answer patterns may be found in the **Results for objective 4 **section.

In our evaluation, we discarded answer patterns only occurring once, and tallied up all remaining patterns. We refer to our top 10 grouped patterns as "common" patterns. Negations occurred infrequently in the answer passages; therefore they are not represented in our results for this objective.

## Results

Our initial aims were to determine how well translational research concepts are covered in the UMLS Metathesaurus and how well MM functions as a concept-identification tool within the domain of genomic medicine. The goals of our later objectives were to determine the location of answers within a FT article and to identify common answer patterns. Results by objective are described below.

### Result for objective 1

Related to our first objective, we found that mutation types were not covered in the UMLS Metathesaurus. Due to this finding, we did not include the first question (<201> What [MUTATIONS] in the Raf gene are associated with cancer?) in our UMLS evaluation and MM assessment.

**Figure 1:**
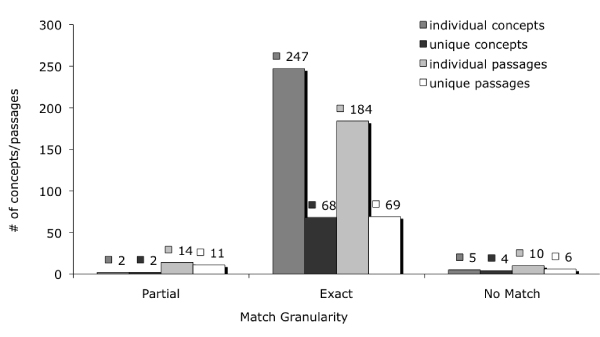
**UMLS matches for questions 216, 218 and 224**. UMLS coverage of gold standard individual concepts, unique concepts, individual passages and unique passages that are associated with questions <216>, <218>, and <224>.

For a given question, answer concepts from our gold standard data set often appeared more than once. For example, in our answer set for <218>, there were 37 answer passages with the concept CB1 (CANNABINOID RECEPTOR). Therefore, in our evaluation, we distinguish between *individual concepts *and *unique concepts*. Our evaluation of ***individual concepts ***includes all concepts even if they appear in multiple answer passages. Where as with ***unique concepts***, answer concepts may only be counted once. In addition, for each answer passage, there may be more than one concept associated with an answer passage. We count *individual passages *and *unique passages *as the sets of concepts associated with answer passages. Our total number of ***individual passages ***includes all sets of concepts, even if the set appears in multiple answer passages. Only unique sets of concepts are counted in our evaluation of ***unique passages***. Figure 1 illustrates the match granularity (partial, exact or no match) of gold standard individual concepts, unique concepts, individual passages and unique passages that are associated with questions <216>, <218>, and <224>.

Overall, we found the UMLS coverage of answers to questions of forms similar to the later three questions to be very good. Approximately 92% of the unique answer concepts and 80% of unique answer passages had matches in the UMLS Metathesaurus.

### Result for objective 2

**Figure 2:**
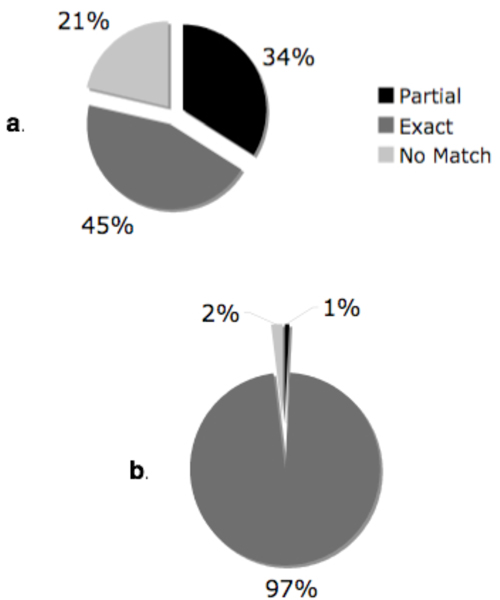
**MetaMap access (a) vs. UMLS coverage (b)**. Pie charts to compare MetaMap's access to individual translational research concepts and UMLS coverage of these concepts.

**Figure 3:**
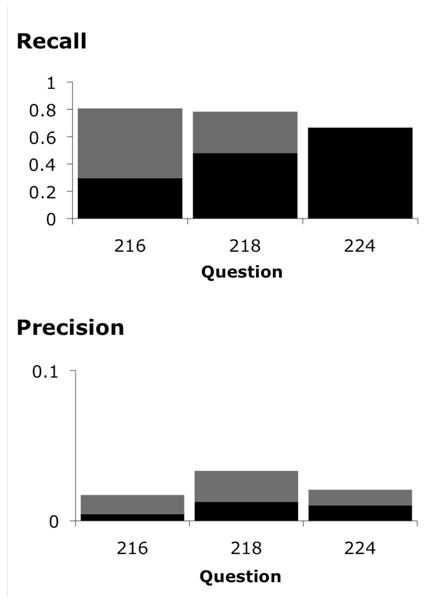
**MetaMap precision and recall calculations**. MetaMap precision and recall calculations for questions <216>, <218>, and <224>. The dark columns take only exact matches into consideration. The light columns take both partial and exact matches into consideration.

**Figure 4:**
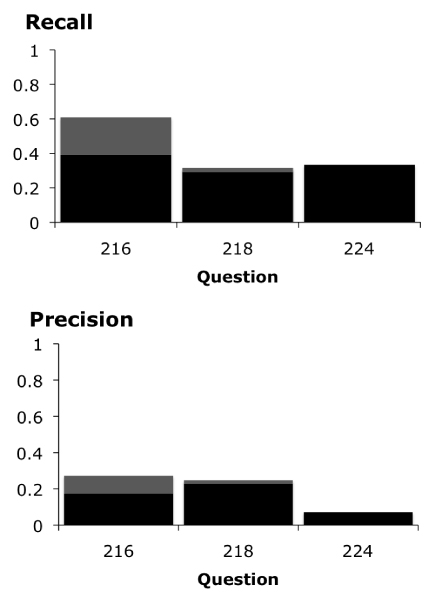
**Baseline precision and recall calculations**. Baseline algorithm precision and recall calculations for questions <216>, <218>, and <224>. The dark columns take only exact matches into consideration. The light columns take both partial and exact matches into consideration.

We compared MM's access to translational research concepts to UMLS coverage of these concepts. We found that MM identified 46% of the answers covered in the UMLS (See Figure 2). In further evaluation, we compared MM's ability to identify answer concepts to our baseline algorithm using precision/recall measures (Figures 3 &4).

The recall values of MM for questions <216>, <218> and <224> were higher than that of the baseline algorithm, although not statistically significant (*P *= 0.068). These results suggest that we may need to explore ways to improve MM's access to translational research concepts. One approach would be adjusting filtering options. Some gene names may be suppressed by the MM program because it is most often used in applications such as RIDeM, where the user is primarily interested in answering clinical questions. Additionally, it may be possible to improve MM's access by creating a customized view for the genomic medicine domain. The Lister Hill NLP Content View (LNCV) Project is currently creating such customized views for other areas of research [14].

### Result for objective 3

**Figure 5:**
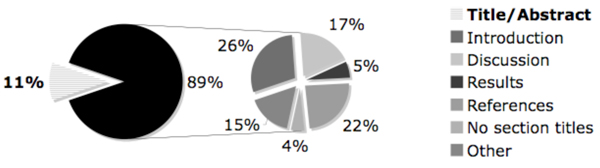
**Answer passage location**. Pie chart representation of the location of answers to translational research questions within full-text articles.

In evaluating the location of answers to translational research questions within the FT documents, we found that answers were located primarily in areas other than the title and abstract (See Figure 5). Only 11% of the answers were found in the title or abstract. This is in contrast with answers to clinical questions, where as in the case of the RIDeM project, the majority of the answers may be found in MEDLINE abstracts. In the case of answers to translational research questions, answers were found most often in the Introduction section, accounting for 26% of the answer passages.

### Result for objective 4

Finally, we identified some common patterns that exist in answer passages. Table 1 lists our top 10 ranked patterns. Examples for common pattern components are shown in Table 2. Examples for common relationships captured in answer patterns are as follows:

**Table 1:** Top ranked answer patterns.

**Top pattern groups**	**Number of occurrences**	**Question(s) represented**
GeneName ASSOCIATED_WITH PrimaryCondition in [SubjectType]	31	216, 218, 224
GeneName MutatorType/MutationType AFFECTS PrimaryCondition in [SubjectType]	21	201, 218
GeneName AFFECTS PrimaryCondition in [SubjectType]	13	216, 218
GeneName OBSERVED_IN PrimaryCondition in [SubjectType]	12	201, 218
MutationType/EnvironmentalCondition of GeneName CAUSES PrimaryCondition in [SubjectType]	11	216, 218
MutationType in GeneName HAS PrimaryCondition	9	216, 218
GeneName MutationType/MutatorType in GeneName/MutationName OBSERVED_IN SubjectType PrimaryCondition	9	201, 216
AFFECT_OF GeneName [in EnvironmentalCondition] on PrimaryCondition [SubjectType]	8	218
EVIDENCE_OF ASSOCIATION_BTWN GeneName and PrimaryCondition in [SubjectType]	5	218
EVIDENCE_OF GeneName in PrimaryCondition	5	218

Top 10 answer patterns occurring in the document set. Square brackets indicate optional entities.

**Table 2:** Common answer pattern components.

**Pattern component**	**Examples**	**Represented UMLS semantic types**
GeneName	BRAF; LHR; luteinizing hormone receptor; GPCRs; CB1 receptor; cannabinoid receptor; NMDA glutamate receptor; CD117	[Gene or Genome] [Amino Acid, Peptide, or Protein] [Receptor]
MutationType	B-RafV599E; V599EB-Raf; BRAFV599E; T1796A mutation; Point mutations; homozygous mutations; null mutation	[Gene or Genome] [Genetic Function] [Cell or Molecular Dysfunction]
MutatorType	antagonist, SR147778; antagonist, rimonabant (SR141716); agonists, CP-55,940 and WIN-55,212-2; glycine-site antagonists	[Organ or Tissue Function] [Organic Chemical] [Amino Acid, Peptide, or Protein] [Pharmacologic Substance] [Biologically Active Substance] [Molecular Function]
EnvironmentalCondition	paraventricular hypothalamic nucleus	[Body Part, Organ, or Organ Component]
PrimaryConditon	melanomas; papilary thyroid cancer; hypogonadism; precocious puberty syndromes; ethonol consumption; myeloid leukemia; germ cell tumors	[Neoplastic Process] [Disease or Syndrome] [Physiologic Function]
SubjectType	human; Childhood; male patient; healthy boy; rats and mice	[Human] [Temporal Concept] [Population Group] [Patient or Disabled Group] [Age Group] [Mammal]

Examples for common answer pattern components and represented UMLS semantic types.

• **ASSOCIATED_WITH**: significantly related to, implicated in, associated with, maps to

• **OBSERVED_IN**: was identified in, were found in, have been detected in, has been described in, were first reported in

• **AFFECTS**: decreased, increased, diminishes, differentially affects, reduces, code for

• **CAUSES**: causes, leads to, is thought to be responsible for, promoted, resulted in

• **HAS**: displayed, reported, is reflected in, demonstrate

• **AFFECT_OF**: innate differences of, reduction of, effects of, stimulation of

• **EVIDENCE_OF**: reported that, our data suggest that, our results have identified, additional studies have confirmed that

It is evident from our observations that the degree of certainty in answers differs. Additionally, gene names and mutation types are often paired. In the future, we will need to examine more question answer pairs in order to refine and validate the identified patterns.

## Discussion

Revisiting our original question of whether NLM tools will be useful for extracting answers to translational research questions, our answer is yes. It is clear from our evaluation that both the UMLS Metathesaurus and the MM program would be useful for identifying translational research terms. However, since the majority of the answers are found in areas other than the title and abstract of FT documents, Natural Language Processing (NLP) and IE techniques are needed to find and extract answers from biomedical text.

In this research, we encountered some limiting factors that are areas for further research and may be taken into consideration when developing NLP and IE algorithms. For example, although we were able to maximize MM recall by using the same UMLS semantic types for all questions, this may also be viewed as a limiting factor because it could partially explain low MM precision. Therefore, in addition to exploring MM filtering options described in the **Result for objective 2 **section, it may also be useful to explore the use of individual semantic types appropriate for each question type.

Another requirement for developing NLP and IE algorithms for answering translational research questions is illustrated in the **Result for objective 1 **section. Our finding that certain mutation types (such as those representative of answer concepts for question <201>) are not covered in the UMLS, suggests a need to explore coverage of mutations in other resources such as The Human Gene Mutation Database [15].

Other limitations in our work are due to the need for a specific translational research NLP challenge. Although we found the data produced in the 2007 TREC Genomics track evaluations to be valuable in this research, we were only able to make use of a small portion of the questions considered in this challenge. This need is further exemplified in that TREC no longer holds a Genomics track evaluation, and other related efforts do not focus specifically on translational research. Such related ongoing challenges include the i2b2 Shared-Task and Workshop Challenges in Natural Language Processing for Clinical Data Medical Extraction [16] and the BioCreAtIvE (Critical Assessment of Information Extraction systems in Biology) challenge evaluations [17].

Furthermore, although the patterns we identify in Objective 4 of our evaluation will be useful for developing NLP and IE algorithms, the results of a translational research NLP challenge will provide the greater number of question answer pairs needed to identify answer patterns for developing these algorithms. As patterns are established, it may be useful to explore the extraction of identified relationships between translational research concepts with SemRep [18], an NLP system that relies on semantics and domain knowledge contained in the UMLS.

## Conclusion

Our results suggest that further exploration of question answering within the context of genomic medicine could contribute to a move from the current reactive mode of medical practice to a P4 Medicine model of practice. By addressing the limitations we discuss in this article and with proper use of NLP and IE techniques that leverage NLM tools, we may be able to extract knowledge reported in biological literature and facilitate question answering in the practice of genomic medicine.

## List of abbreviations used

BioCreAtIvE: Critical Assessment of Information Extraction systems in Biology; FT: Full-text; i2b2: Informatics for Integrating Biology & the Bedside; IE: Information Extraction; LNCV: The Lister Hill NLP Content View; MM: The MetaMap Program; NLP: Natural Language Processing; P4 Medicine: A predictive, personalized, preventative and participatory model of medical practice; RIDeM: Repository for Informed Decision Making; TREC: Text Retrieval Conference; UMLS: Unified Medical Language Systems

## Competing interests

The authors declare that they have no competing interests.

## Authors' contributions

DDF and CLO came up with the concept and design of the approach taken. DDF developed and coded answer and passage extraction and the baseline system. PTH provided advice and critical feedback for the study. CLO conducted the evaluation and prepared the manuscript. All authors participated in draft revisions. All authors contributed to, read and approved the final manuscript.
